# An Epidemiological Study of Cut Throat Injury During COVID-19 Pandemic in a Tertiary Care Centre

**DOI:** 10.1007/s12070-020-02239-4

**Published:** 2020-10-23

**Authors:** Souvagini Acharya, Rajat Kumar Dash, Aurobinda Das, Madhusmita Hota, Chittaranjan Mohapatra, Sarita Dash

**Affiliations:** 1 Dept of ENT, VIMSAR Burla, Sambalpur, Odisha India; 2 DHH, Sambalpur, India

**Keywords:** Cutthroat, Suicidal, Homicidal, Accidental, Predisposing factor

## Abstract

To find out incidence, prevalence, various modes and pattern of cutthroat injury during COVID-19 pandemic and compare with prior non pandemic period. To find out the various factor influencing the suicidal cutthroat and establish the temporal association of suicidal cutthroat with COVID-19 pandemic. It is a retrospective study of cutthroat patients who were managed in ENT Dept. VIMSAR, Burla, Sambalur, Odisha, India from 1st September 2019 to 31st August 2020. Source of information are casualty, IPD and OT registers and online data. Total cases were divided into group-A (prior to COVID-19 pandemic) and group-B (during COVID-19 pandemic) and analysed. Total 24 cutthroat injury cases were treated over 1 year in department of ENT, VIMSAR, Burla, which was 0.054% of total cases attended at casualty and 2.371% of total IPD patients treated. In GROUP-A, total 10 cases with M:F = 9:1,suicidal 4 cases (40%), homicidal 6 cases (60%), and no accidental cases were recorded. While in GROUP-B, total 14 case with M:F = 14:0, suicidal 9 cases (64.28%), homicidal 3 cases (21.42%) and accidental 2 (14.28%) cases were recorded. In our study majority of cases were male with M:F = 23:1. Common age group belongs to 20–30 years with LSES and farmer by occupation. Zone II injury had incidence of 70.83%. Homicide cases proportionally high during non-COVID period while suicide cases high during COVID-19 pandemic. Association of COVID-19 pandemic with suicidal cut throat injury is seems to be significant. Among predisposing risk factors for suicidal, depression during COVID-19 pandemic had seen in 53.84% of total suicidal cases. Incidence and prevalence of Cut throat injury is comparatively high in western odisha which again increases during months of July and August parallel to COVID-19 pandemic. The common mode of cutthroat injury is homicidal, which suddenly changes to suicidal during COVID-19 pandemic. More vulnerable groups were young unemployed male, farmers and labours. Cutthroat injury cases definitely increases during COVID-19 pandemic with most common mode of injury being suicidal attempt, which may be due to economical and psychological imbalances, due to loss of job and fear and social stigma for COVID-19 diseases.

## Introduction

Now a day’s Cut throat injury cases are most often encountered in causality in serious condition. Cut throat wounds are a well-recognized method of homicide, less commonly used mode of suicide and are rarely accidental [1]. The neck contains of vital structures like great vessels, trachea and oesophagus, the injury to which make the cut throat patients to present to causality in critical condition as emergency case [2]. Cut throat injuries poses a great surgical challenge because multiple vital structure are vulnerable to injuries in the small, confined unprotected area [3]. Cut throat injuries are one of the emergency condition managed by ENT specialists [4]. The mode of cut throat injuries can be broadly divided into suicidal, homicidal and accidental [5]. In suicidal attempts family problems, psychiatric illnesses, poverty and love failure are triggering factors while that for homicidal cut throat injuries familial and property related disputes, political conflicts and sex related crimes are common [6]. Accidental causes are mostly related to the road traffic accident and fall injuries. In suicidal cut throat injury tentative cut are have diagnostic value. Immediate danger of cut throat injuries is respiratory obstruction and haemorrhage [7]. Tracheostomy should be performed immediately when airway obstruction or chances of aspiration of blood exists [8]. The depth of injury varies from skin and superficial tissue injury to opening of airway and major blood vessels injury. In all patients cut throat injuries were assessed by assigning the injury to three anatomical zones of neck. Zone I extends from the cricoid cartilage to clavicle. Zone II is between cricoid cartilage and angle of mandible. Zone III is between angle of mandible and base of skull [4]. Tentative cuts i.e. multiple superficial cut marks are diagnostic of suicidal cut throat injury. Psychiatrist provides adequate support and care after surgical treatment [9]. Majority of cases were males with history of psychiatric illness and drug abuse. More vulnerable groups were young unemployed male, farmers and labours and belong to low SES.

## Aims and Objectives

To find out incidence, prevalence, various modes (homicide, suicide and accidental) and pattern (zones and extents) of cut throat injury during COVID-19 pandemic and compare with prior non pandemic period. To find out the various factor influencing the suicidal cut throat injury and establish the temporal association of suicidal cutthroat injury to COVID-19 pandemic.

## Material and Methods

It is a retrospective study of cut throat injury patients who were managed in ENT Dept. VIMSAR, Burla, over a period of 1 year i.e. from 1st September 2019 to 31st August 2020. We divides into two groups, group-A i.e. cases from 1st September 2019 to 28th February 2020 (During non COVID period) and group-B i.e. cases from 1st March 2020 to 31st August 2020 (During COVID pandemic period). Source of information are casualty register, IPD register, OT register and online data. Total 24 numbers of cases were recorded and analysed with detail history, epidemiological parameter, and clinical presentation. Detail history of substance abuse, family dispute and psychiatric problem had taken. Suicidal cutthroat injuries identified by tentative cut mark (Fig. 6). The data extracted for analysis were age, sex, occupation, SES, mode of injury, clinical presentation, anatomical zone with extent of injuries and outcomes.

## Observation

Total 24 cut throat injury cases were treated over a period of 1 year, Which is 0.054% of total cases attended at casualty and 2.371% of total IPD patients treated in the department of ENT, VIMSAR, BURLA. Prior to COVID-19 pandemic incidence were 0.041% and 1.455% of total number of patient attended at casualty and total number of IPD patients treated respectively which is increases to 0.068% and 4.307% during COVID-19 pandemic, which is shown in Table. 1.

**Table. 1:** Showing incidence of cut throat injury cases

Period	Cut throat cases	Total no of pt attended at casualty	Percentage (%)	Total no of IPD patients	Percentage (%)
01/09/2019–31/08/2020	24	44,518	0.054	1012	2.371
01/09/2019–28/02/2020 (Group-A)	10	24,067	0.041	687	1.455
01/03/2020–31/08/2020 (Group-B)	14	20,451	0.068	325	4.307

**Fig. 1:**
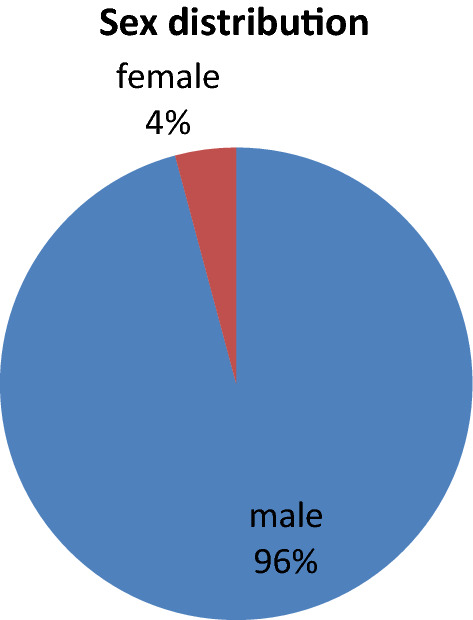
Showing sex distribution of cut throat injury cases

**Fig. 2:**
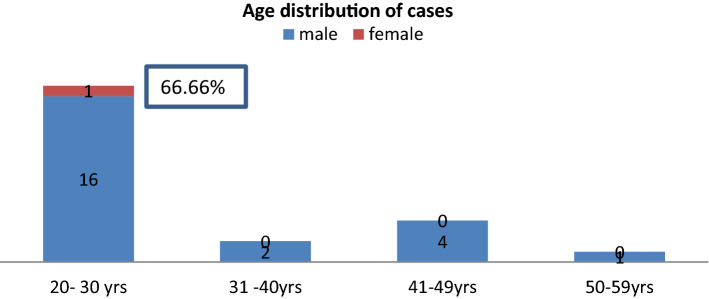
Showing age distribution of cut throat injury cases

In our study, out of 24 cases, 23 i.e. 95.83% are male and 1 i.e. 4.16% are female with M:F = 23:1. Common age group affected were 20–30 year, which is shown in bar diagram below (Figs. 1, 2).

**Fig. 3:**
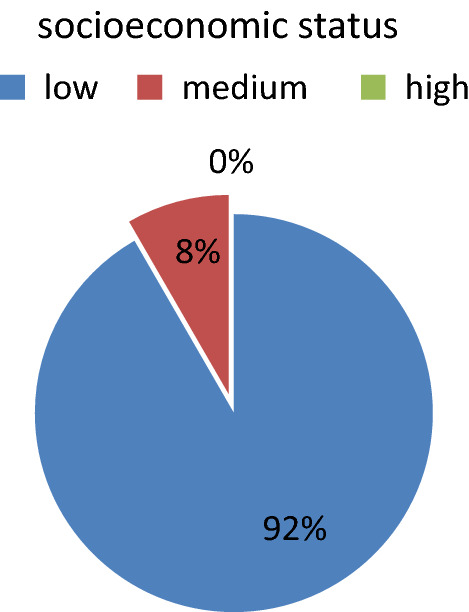
SES distribution of of cut throat injury cases cases

Highest incidence of cutthroat injury cases were belongs to low socioeconomic status, farmers and labour. (Table. 2 and Fig. 3).

**Table. 2:** Occupational distribution of of cut throat injury cases

Occupation	No of cases	Percentage (%)
Labour	08	33.33
Farmer	12	50
Service (private company)	02	8.33
Student	02	8.33

**Fig. 4:**
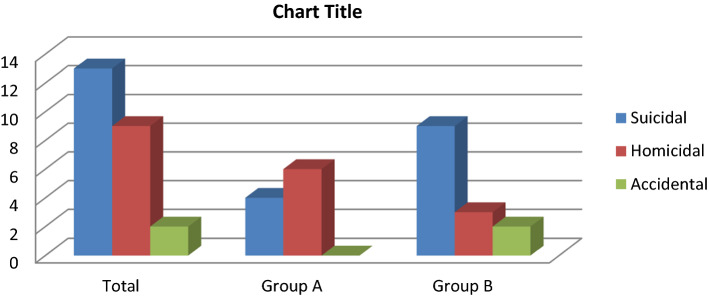
Showing comparison of mode of cut throat injury

Among various modes of injury, Homicide cases proportionally high during non-COVID period constituting 60% cases followed by suicidal cases constituting 40% cases. Where as suicide cases high during COVID pandemic constituting 64.28% cases followed by homicide cases constituting 21.42% cases and accidental constituting 14.8% cases. Overall within 1 year, suicidal cases constituting 54.16% cases followed by homicide cases constituting 37.5% cases and accidental constituting 8.33% cases, which is shown in Table. 3 The trend of homicidal modes changes to suicidal during COVID-19 pandemic. Association of COVID-19 pandemic with suicidal cut throat injury is seems to be significant (Fig. 4).

**Table. 3:** Showing various modes of cut throat injury cases

Modes	Homicidal		Suicidal		Accidental	
Duration	Number	Percentage (%)	Number	Percentage (%)	Number	Percentage (%)
Overall 1 yr	9	37.5	13	54.16	2	8.33
Group-A	6	60	4	40	0	0
Group-B	3	21.42	9	64.28	2	8.33

Among the various predisposing risk factors for suicidal cut throat, depression due to COVID-19 pandemic had seen in highest cases, constituting 53.84% of cases followed by Psychiatric illness (23.7%), family problem (15.38%) and substance abuse (7.69%), shown in Table. 4. Depression may be due to economical and psychological imbalance during COVID-19 because of loss of jobs, fear of COVID-19 disease, anxiety, loss hope of living, detached from near to dears and social stigma.

**Table. 4:** Risk factors for suicidal cut throat injury cases {total 13}

Types of risk factors	No of cases	Percentage (%)
Substance abuse	01	7.69
Psychiatric problem	03	23.07
Family problem	02	15.38
Depression (due to COVID-19)	07	53.84

**Fig. 5:**
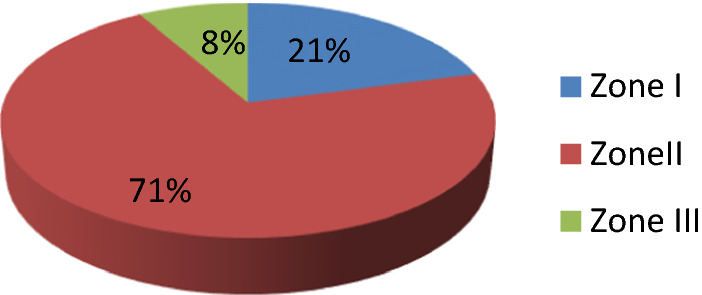
According to site of injury of cut throat cases

**Fig. 6:**
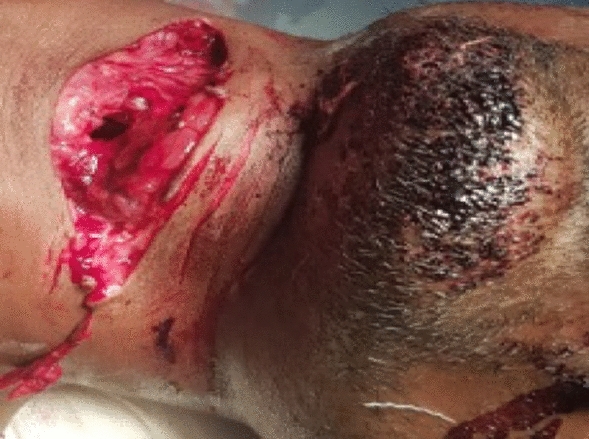
Suicidal cut injury showing tentative cuts

In our study, according to site of cut throat injury zone II injury had highest incidence constituting 71% of cases followed by zone-I (21%) and zone-III(8%), which is shown in Figs. (5, 6).

**Fig. 7:**
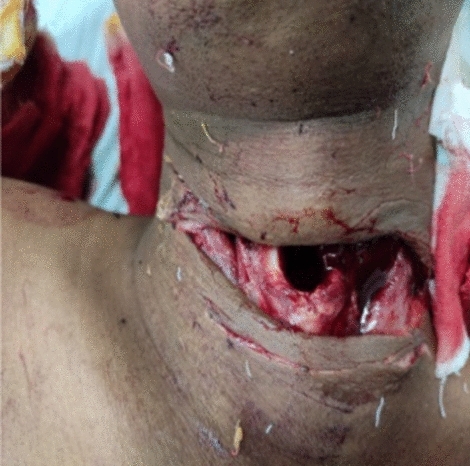
Showing open airways and tentative cuts

**Fig. 8:**
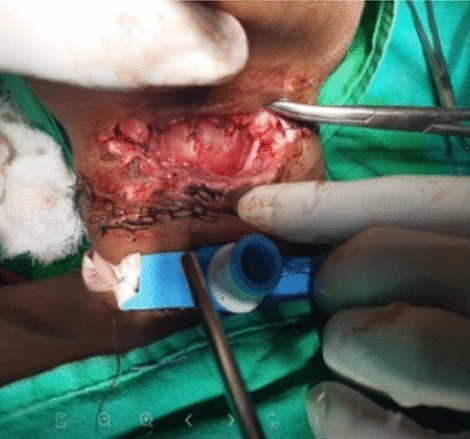
Showing trachestomy

 In our study, majority (54.16%) of cut throat injury were found to be deep with opened airway had required tracheostomy first to secure airway before definite repair (Figs. 7, 8). Airway injury found at the level of thyrohoid membrane and cricothyroid membrane region, thyroid cartilage injury also found in few cases. Oesophagus injury found in one case and external carotid artery injury found in one case shown in Table. 5. All cases are received at causality in critical condition, some cases received in shock. Haemostasis and hemodynamic stability maintained first and surgical repair was done. Postoperative wound care had taken. All cases are cured and discharged except one case, in which death occurred in COVID care Hospital in fourth post operative day having healthy wound site. He attempted suicide after knowing his COVID-19 status and had open airway and have cough and fever.

**Table. 5:** According to extension of injury of cut throat cases

Extension of injury	Number of cases	Percentage (%)
Skin subcutaneous tissue, muscle	09	37.50
Air way open	13	54.16
Major vessel injury	01	4.16
Oesophagus injury	01	4.16

**Fig. 9:**
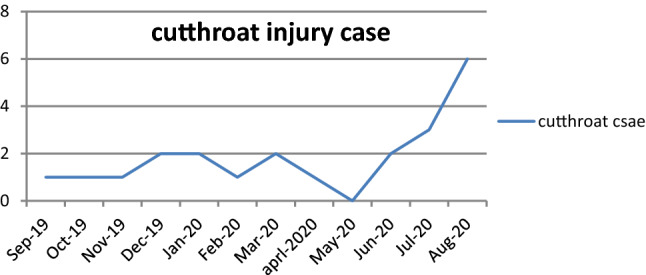
Cutthroat injury incidence according to month

**Fig. 10:**
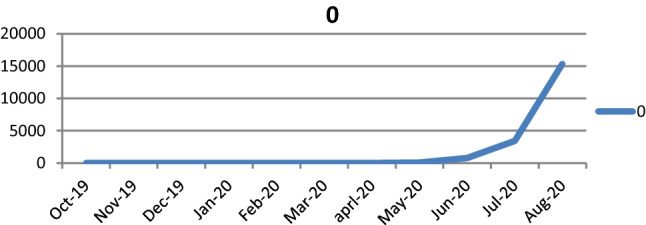
COVID-19 incidence according to month

The incidence of cut throat injuries suddenly increase in June, July and August (Fig. 9), which is shows parallelism with incidence of COVID-19 cases (Fig. 10). So there is temporal association of suicidal cutthroat with COVID-19 pandemic.

## Discussion

In our study, incidence of cut throat injury was 0.054% of total patient attended to casualty and 2.371% of total IPD patient. Majority of cases i.e. 23 (95.83%) were male and 1 (4.16%) was female with M:F ratio of 23:1,which is similar to study done by Aich M et al. [10]. The majority of victims i.e. 66.66% were young adults between 20 and 30 years of age, which is similar to study done by Debdula Chakraborty et al. [4]. Majority of cut throat injury cases were belongs to low SES constituting 92%, which is similar to study done by Debdula Chakraborty et al. [4]. Majority of cases belongs to farmer (50%) and labour (33.33%) by occupation.

In our study, overall most common mode of cut throat injury was suicidal, which is contradict to study done by Modi and Pandy [11] where homicidal is most common mode. However prior to COVID-19 pandemic homicidal was the common mode of cut throat injury. The incidence of cut throat injury was comparatively high during months of July and August, which was parallel to incidence of COVID-19. Among the various predisposing risk factors for suicidal cut throat, depression related to COVID-19 pandemic had seen in most of cases, constituting 53.84% of cases followed by Psychiatric illness constituting 23.7%, family problem constituting 15.38% and substance abuse constituting 7.69%. The trend of homicidal cut throat suddenly changes to suicidal during COVID-19 period, which may be due to psychological and economical imbalance during COVID-19 pandemic, due to loss of job, social stigma, fear for disease, restriction of social activity, home isolation and domestic violence.

Majority of cases (54.16%) of cut throat injury were found to be deep with opened air way and required tracheostomy prior to repair, which is similar to study done by Debdula Chakraborty et al. [4]. The predominance of Zone II injuries in our study is attributable to the fact that unlike Zones I and III, Zone II is not protected by bony structures making it more vulnerable to injuries, which is similar to study done by Duncan JAT et al. [12].

Most of cases of cut throat injury were received at causality in critical condition even some cases in haemorrhagic shock and compromised airway. However prompt management by proper clinical examination and assessment of hemodynamic stability with maintaining Haemostasis and securing airway by surgical repair and proper Postoperative wound can save life of patient with good outcome.

## Conclusion

Most patients of cut throat injury are young adolescence male of age group of 20–30 years and belongs to low SES. Cut throat injury cases definitely increases during COVID-19 pandemic with most common mode of injury being suicidal attempt, which may be due to economical and psychological imbalance during COVID-19 pandemic, due to loss of job, fear of COVID-19 disease, anxiety, loss hope of living, detached from near to dears and social stigma. Prompt management with securing air way, maintaining haemostasis and proper post operative care decrease morbidity and mortality of cut throat injury cases. Social awareness, counselling, proper education and creation of opportunity for job may reduce the incidence of suicidal cut throat injury.

## Limitation of Our Study

Duration of study is 1 year with small sample size and COVID-19 pandemic is not yet over.

## Abbreviations

*ENT*: Ear nose and throat
*LSES*: Low socio economical status
*IPD*: Indoor patient department
